# Letter to the Editor From Finsterer “Diverse Phenotypes of Mitochondrial Disease With Varying Levels of Heteroplasmy”

**DOI:** 10.1210/jcemcr/luaf127

**Published:** 2025-07-01

**Authors:** Josef Finsterer

**Affiliations:** Neurology Department, Neurology & Neurophysiology Center, Vienna 1180, Austria

To the Editor—

I was interested to read the article by John et al about a 42-year-old woman who had been suffering from recurrent pancreatitis and consecutive diabetes mellitus for 4 years [1]. The family history was positive for diabetes (mother, brother, aunt, uncle), hypoacusis (mother), heart disease (brother), and early death (mother, brother, daughter, aunt) [1]. Genetic testing revealed the common variant m.3243A > G in MT-TL1 with a heteroplasmy rate of 15% in the blood [1]. The patient received pancreatic enzyme supplements, a pancreatic duct stent, and insulin therapy [1]. It was concluded that the m.3243A > G variant exhibits phenotypic heterogeneity and that first-degree relatives of m.3243A > G carriers should be screened for the mutation [1]. The study is noteworthy, but several points should be discussed.

The first point is that phenotypic variability in mitochondrial disorders resulting from mtDNA mutations is not only due to variable heteroplasmy rates, but also to tissue distribution, mtDNA copy number variation, haplotype, mtDNA polymorphisms, and mutations in nuclear genes involved in mitochondrial morphology, function, and metabolism [2]. Was the mtDNA copy number determined in the index patient and her daughter? To which haplotype did the index patient belong? Was whole-exome sequencing performed to determine whether additional nuclear mutations altered the phenotype?

The second point is that the family history was positive for early death (mother, brother, daughter), but the index patient was not prospectively and systematically screened for possible cardiac involvement, in particular hypertrophic cardiomyopathy or malignant arrhythmias, which are common phenotypic manifestations of m.3243A > G carriers [3]. To minimize the risk of sudden death even in the index patient, it would be necessary to exclude all risk factors for sudden death.

The third point is that elevated serum lactate is often associated with elevated cerebrospinal fluid lactate. This is particularly the case in m.3243A > G carriers who manifest with stroke-like episodes [4]. Was there any evidence of elevated cerebrospinal fluid lactate in addition to the elevated serum lactate? Cerebral acidosis should be recognized as it can be a risk factor for strokes and seizures [5].

The fourth point is that no cerebral imaging of the index patient was reported [1]. Did the index patient also have calcification of the basal ganglia, a common finding in MELAS (mitochondrial encephalopathy, lactic acidosis, and stroke-like episodes) patients [6].

The fifth point is that the index patient's daughter was not autopsied [1]. An autopsy could have clarified the cause of the “blooming” in the globus pallidus on magnetic resonance imaging. Was the blooming due to calcification or iron deposition?

Finally, the expression “recurrent episodes of myopathy” is not understandable. Do the authors mean that there were recurrent episodes of muscle weakness, but not persistent muscle weakness, as in periodic paralysis or myasthenia? Was there evidence that the index patient's daughter had a different condition from her mother?

In summary, carriers of m.3243A > G need to be prospectively screened for multisystem involvement and all their first-degree relatives should be screened for phenotypic and genotypic features of a mitochondrial disorder.

## Disclosure

The author declares that the research was conducted in the absence of any commercial or financial relationships that could be construed as a potential conflict of interest.

## Data Availability

All data are available from the corresponding author.
